# Quinolone Prophylaxis in Transrectal Ultrasound Guided Prostate Biopsy: An Eight-Year Single Center Experience

**DOI:** 10.1155/2013/452107

**Published:** 2013-12-23

**Authors:** Bing-Juin Chiang, Yeong Shiau Pu, Shiu-Dong Chung, Shih-Ping Liu, Hong-Jeng Yu, Shuo-Meng Wang, Hong-Chiang Chang, I-Ni Chiang, Chao-Yuan Huang

**Affiliations:** ^1^Department of Urology, National Taiwan University Hospital, College of Medicine, National Taiwan University, No. 7, Chung Shan S. Road (Zhongshan S. Road), Zhongzheng District, Taipei City 10002, Taiwan; ^2^Department of Urology, Cardinal Tien Hospital, College of Medicine, Fu Jen Catholic University, No. 362, Zhongzheng Road, Xindian District, New Taipei City 231, Taiwan; ^3^Division of Urology, Department of Surgery, Far Eastern Memorial Hospital, No. 21, Section 2, Nanya S. Road, Banqiao District, New Taipei City 220, Taiwan; ^4^Department of Urology, Tzu Chi General Hospital, No. 707, Section 3, Chung Yang Road, Hualien 970, Taiwan

## Abstract

We retrospectively evaluated the efficacy of prophylaxis with pipemidic acid and levofloxacin in transrectal ultrasound guided prostate biopsy (TRUSP-Bx). From January 2002 to December 2004, patients receiving oral pipemidic acid 500 mg twice daily for three days with or without a preoperative intravenous cefazolin 1 gm injection comprised group A. Between January 2005 and December 2009, patients receiving oral levofloxacin 500 mg one hour before biopsy comprised group B. We calculated the annual febrile urinary tract infection (fUTI) rates. Patients' characteristics, including age, prophylactic antibiotics, biopsy core numbers, pathologic results, PSA, and the spectrums and susceptibility of pathogens, were also evaluated. A total of 1313 (35.5%) patients belonged to group A, while 2381 (64.5%) patients belonged to group B. Seventy-three patients experienced postoperative infectious complications. There was a significant difference in the fUTI rate between groups A and B (3.7% versus 1.0%, *P* < 0.001). The yearly fUTI rates varied from 0.6 to 3.9% between 2002 and 2009. Of the 73 patients with fUTI, those receiving levofloxacin prophylaxis were more likely to harbor fluoroquinolone-resistant pathogens (*P* < 0.001). *E. coli* was the most common pathogen in both groups. Levofloxacin remains effective and appears superior to pipemidic acid based prophylaxis.

## 1. Introduction

Although antibiotic prophylaxis generally reduces the risk of infectious complications following transrectal ultrasound guided prostate biopsy (TRUSP-Bx), wide variability in antibiotics prophylaxis has been reported [1]. Oral quinolones were the most common prophylactic antibiotics (91.6%) either alone or in combination with another antibiotic. Quinolones are the most active agent against aerobic gram-negative bacilli and accumulate in prostate, feces, and urine. They are commonly used in treatment of urinary tract infection (UTI) and prophylaxis in urologic surgery [2]. The European Association of Urology (EAU) guidelines on prostate cancer state that “quinolones are the drug of choice before TRUSP-Bx” [3]. However, there is no consensus with regard to the prophylactic antibiotics in Taiwan [4]. Pipemidic acid, an agent structurally related to nalidixic acid, was first published in 1974 [5]. It used to be one of the prophylactic antibiotics in TRUSP-Bx in our institute until 2004. In 2002, Griffith et al. conducted a study using a single dose of levofloxacin (500 mg) as prophylaxis for prostate biopsy and reported a low overall infection rate [6]. According to these results, antibiotic prophylaxis regimens for TRUSP-Bx were unified in our institute since January 2005, when we adopted this simple and convenient regimen. In the current study, we shared our experiences of quinolone prophylaxis including pipemidic acid and levofloxacin between 2002 and 2009. The febrile UTI rate following TRUSP-Bx the spectrums and susceptibility of pathogens were evaluated.

## 2. Materials and Methods

We retrospectively reviewed the medical records of patients who received TRUSP-Bx from a single institution, National Taiwan University Hospital (NTUH), between January 2002 and December 2009. Indications for biopsy included an increased PSA level and/or abnormal digital rectal examination. Patients who underwent other surgeries concurrently and those with a preexisting diagnosis of prostate cancer were excluded.

All patients received rectal preparation with two tablets of bisacodyl suppositories the day before biopsy. Between January 2002 and December 2004, patients receiving oral pipemidic acid 500 mg twice daily for three days with or without a preoperative intravenous cefazolin 1gm injection were enrolled in group A. From January 2005 to December 2009, patients receiving levofloxacin 500 mg in a single dose one hour before biopsy were enrolled in group B. Digital rectal examination (DRE) was performed immediately before the procedure to avoid a transfecal biopsy. The operation was terminated if DRE revealed any residual stool. Subsequently, rectal disinfection with an iodine swab was performed four times. Transrectal ultrasound (TRUS) examinations were performed with a real-time ultrasound scanner (B&K 1846 model) using a 7-MHz transducer. Biopsies were taken with 18G Tru-cut biopsy needles during longitudinal scanning. Either a sextant or a ≥12-core biopsy (with or without an additional specific lesion biopsy) was performed according to each physician's preference. The biopsy can be done either in outpatient clinic setting or inpatient setting with additional one-day hospitalization according to each patient's preference.

Definition of febrile UTI was based on the presence of the following criteria in the medical records: (1) a body temperature above 38°C two weeks after biopsy, (2) new-onset lower urinary tract symptoms (urgency, frequency, and/or dysuria) or acute epididymitis, and (3) the absence of other sources of infection.

We calculated the annual febrile UTI rates. Patients' characteristics, including age, prophylactic antibiotics, biopsy core numbers, pathologic results, and PSA level were assessed. All available blood tests, urine analyses, blood cultures (at least one set in each patient), and urine cultures were also evaluated. However, some data may have been censored. The annual levofloxacin-resistance rate of *E. coli* was retrieved from annual summary documents of the infection control center of NTUH, an academically affiliated hospital providing both primary and tertiary care in northern Taiwan. The retrieved information was provided for discussion. The bacterial isolates and antimicrobial resistance from various clinical specimens collected from both outpatients and inpatients were reported annually. To calculate the resistance rates, isolates of each species recovered from each patient were calculated once within 7 days (no-duplicate isolates). Isolates were classified as susceptible, resistant, or intermediate according to the National Committee for Clinical Laboratory Standards criteria [7].

This study was approved by the institutional review board. Results were analyzed with commercial statistical software, SPSS. Chi-square analysis, median tests, and logistic regression with febrile UTI as the dependent variable were performed. Statistical significance was defined as *P* < 0.05.

## 3. Results

**Figure 1:**
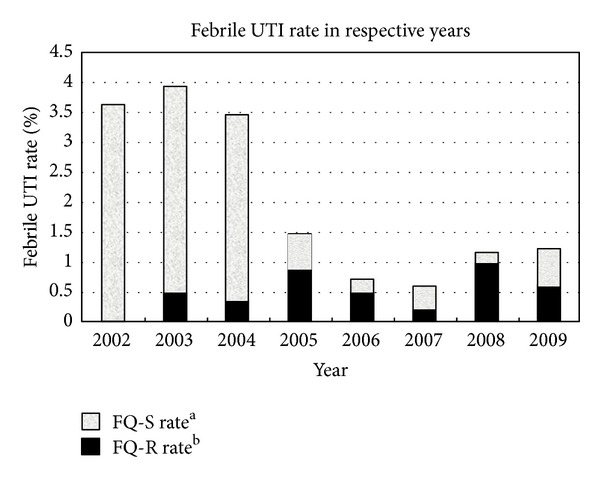
Febrile UTI rate per year of assessment. (a) FQ-S rate: fluoroquinolone-sensitive febrile UTI rate. (b) FQ-R rate: fluoroquinolone-resistant febrile UTI rate. The infection (febrile UTI) rates (%) were 3.6, 3.9, 3.5, 1.5, 0.7, 0.6, 1.2, and 1.2 in 2002, 2003, 2004, 2005, 2006, 2007, 2008, and 2009, respectively. The FQ-S rates (%) were 3.6, 3.4, 3.1, 0.6, 0.2, 0.4, 0.2, and 0.6 in 2002, 2003, 2004, 2005, 2006, 2007, 2008, and 2009, respectively. The FQ-R rates (%) were 0, 0.5, 0.3, 0.8, 0.5, 0.2, 1.0, and 0.6 in 2002, 2003, 2004, 2005, 2006, 2007, 2008, and 2009, respectively.

A total of 3694 TRUSP-Bx procedures were conducted between 2002 and 2009. The mean age of patients was 67.6 years (range: 22 to 95), while the median PSA level was 10.0 ng/mL (range: 1 to 12960). There was no significant difference between groups A and B in age and PSA level. The individual characteristics of both groups are listed in Table 1. Between 2002 and 2004, 1313 (35.5%) patients underwent biopsies and were enrolled in group A. From 2005 to 2009, 2381 (64.5%) patients underwent biopsies and were enrolled in group B. As outlined in Table 2, the type of antibiotic prophylaxis was the only predictor of febrile UTI in logistic regression analysis (odds ratio, 3.74; 95% CI, 2.26–6.20). A total of 48 patients in group A (48/1313, 3.7%) and 25 patients in group B (25/2381, 1.0%) experienced a febrile UTI. The febrile UTI rates were significantly different between the two groups (3.7% versus 1.0%, *P* < 0.001). When stratified by years, the febrile UTI rates (%) were 3.6, 3.9, 3.5, 1.5, 0.7, 0.6, 1.2, and 1.2 in 2002, 2003, 2004, 2005, 2006, 2007, 2008, and 2009, respectively (Figure 1). Figure 1 also demonstrated the rates of fluoroquinolone-sensitive/resistant UTI during each year. Of the 73 patients with febrile UTI, those receiving levofloxacin prophylaxis were more likely to harbor fluoroquinolone-resistant pathogens (*P* < 0.001). There were no significant differences in annual febrile UTI rates between 2002 and 2004 (*P* = 0.93), or between 2005 and 2009 (*P* = 0.65). Table 1 also demonstrated the clinical presentations of the 73 patients with febrile UTI. Although the rates of pyuria and positive urine culture were significantly different between the two groups, the data might be censored. Most infectious complications following TRUSP-Bx occurred within 2 days of the procedure in both groups. The rate of leukocytosis was 64.3% in group A and 52.6% in group B. The mean length of hospital stay was 6.4 days in group A and 5.0 days in group B. Urine and blood cultures were sampled from all patients with infections complications, and positive cultures were found in 58.3% of group A and 72% of group B. 

An overview of bacterial isolates from blood and urine cultures is listed in Table 3. *E. coli* was the most common pathogen in both groups (58.6% in group A versus 84.2% in group B). As described in Table 4, the susceptibility of *E. coli* strains to ampicillin was only observed among 17.6% of group A and 6.3% of group B. Only 2 (87.5%) of the 16 *E. coli* cultures yielded in group B were susceptible to fluoroquinolones. However, susceptibility to both amikacin and ertapenem was 100% in both groups.

## 4. Discussion

According to the 2010 EAU guidelines on prostate cancer, the cumulative infection rate following TRUS-guided prostate biopsy was 2.5%, which included prostatitis, a fever >38.5°C, and epididymitis [3]. In our study, the febrile UTI rate following TRUSP-Bx decreased from 3.7% to 1% after the prophylactic regimens were changed from pipemidic acid to levofloxacin. In agreement with the EAU guidelines, our results suggest that levofloxacin is an effective prophylaxis for TRUS-guided prostate biopsy. Thus, we confirmed the efficacy of levofloxacin prophylaxis in TRUSP-Bx. To our knowledge, the current study is the largest to evaluate the efficacy of levofloxacin prophylaxis and the only one to evaluate the efficacy of pipemidic acid based prophylaxis in TRUSP-Bx.

The most common pathogen responsible for the febrile UTI following TRUSP-Bx was *E. coli*. The rate of fluoroquinolone resistance was significantly different between groups A and B. With the exception of fluoroquinolone, the resistance patterns of *E. coli* in group B were similar to those observed in annual reports from our institute (Table 4). In 2003, Tal et al. were the first to describe the susceptibility pattern of bacteria isolated from infectious complications after TRUSP-Bx [8]. High rates of resistance to fluoroquinolones were noted in patients receiving fluoroquinolone prophylaxis, which the authors attributed to preexisting fluoroquinolone-resistant strains and bacterial selection. Based on our results, we also support this viewpoint. However, some patients have been noted to suffer from fluoroquinolone-sensitive *E. coli* infection even after fluoroquinolone prophylaxis [8, 9]. Without antibiotic prophylaxis, transient bacteremia seems completely inevitable [10]. We postulated that host factors such as immune status and bacterial load might play a role in this phenomenon.

Since fluoroquinolones have a broad spectrum of activity against most gram-negative organisms and a good prostatic tissue penetration, they are widely used for antibiotic prophylaxis. However, prolonged usage of fluoroquinolones has resulted in increasing microbial resistance [11, 12]. Recent years have shown an increase in resistant *E. coli* [13–17]. Indeed, a trend of increasing resistance to fluoroquinolones among common gram-negative bacteria was also observed in Taiwan [18]. Recent reports have demonstrated an emergence of fluoroquinolone-resistant infections following TRUSP-Bx [8, 19]. Feliciano et al. reviewed the medical records of patients who received gatifloxacin or levofloxacin and experienced infectious complications after TRUSP-Bx from 2004 to 2006 and reported an increase in infectious complications and fluoroquinolone resistance [20]. Lange et al. reported a 0.5% rate of urosepsis among 4749 patients examined between 2001 and 2006 [9]. Unfortunately, these studies did not provide enough information, such as the annual infection rate, rectal preparation, and local resistance patterns. Changing antibiotic prophylaxis from a quinolone-based regimen may result in an increased risk of infectious complication [21]. Thus, periodic reassessment of the efficacy of fluoroquinolone prophylaxis is imperative. Accordingly, we conducted a retrospective study to evaluate febrile UTI rate following TRUSP-Bx between 2002 and 2009. We also provided regional information of annual resistance patterns. Since a number of prior reports revealed that rectal preparation reduces the risk of infectious complications, we performed rectal preparations and digital rectal examinations before TRUSP-Bx for all patients [22–24]. We found that antibiotic prophylaxis was the only independent predictor of febrile UTI risk. Since 2005, we adopted the convenient regimen of a single dose of levofloxacin as prophylaxis. From 2005 to 2009, the febrile UTI rates varied between 0.6% and 1.5%. In our institute, all cultures were evaluated for annual microbial resistance patterns. According to the annual summary documents of the infection control center of NTUH, the *E. coli* resistance rate to levofloxacin is 29.5, 28.0, 26.5, 27.5, and 28.0% in respective years from 2005 to 2009. The secular trend of the febrile UTI rate following TRUSP-Bx (1.5, 0.7, 0.6, 1.2, and 1.2% from 2005 to 2009) seems related to the *E. coli* resistance rate to levofloxacin. Even though microbial resistance is emerging in the literatures, the febrile UTI rate was acceptable in our study. Besides, although prior studies have warned of the impact of fluoroquinolone-resistant Enterobacteriaceae and *Clostridium difficile* on the safety of prostate biopsy, these were not isolated in the present study [21, 25, 26]. Thus, we suggest that fluoroquinolones remain appropriate for use as antibiotic prophylaxis even though around 30% of *E. coli* were resistant to fluoroquinolones based on annual reports. In addition, according to our results, fluoroquinolones are superior to nonfluorinated quinolones in prophylaxis of TRUSP-Bx.

However, due to the increasing worldwide microbial resistance, the major complication rate of TRUSP-Bx is unlikely to remain as low as it has been traditionally [27]. Although the strategy of combining antibiotics for prophylactic purposes has been suggested in the literature [9, 27, 28], the convenience of such regimens may be limited. Since complete elimination of infectious complications is impossible, second- or third-line antibiotics should be reserved for the treatment of infection. Further studies should be conducted to determine which regimen has the best cost-benefit tradeoff. Although long-term antibiotic cycling regimens are suggested in the literature [9, 29], the susceptibility of pathogens to most available antibiotics was also found to decrease with time in annual reports (Table 4). Currently, no single agent is as effective as fluoroquinolones in the prophylaxis following TRUSP-Bx. In addition, antibiotic cycling in TRUSP-Bx only will not eliminate resistance strains unless fluoroquinolones are completely abandoned in a community for a period of time. Indeed, infectious complications result from microbial selection and the microbial resistance patterns in communities. Antibiotic prophylaxis should be applied by individuals. Detailed medical history should be obtained, including recent antibiotic usage, since antibiotics change microbial resistance patterns [30]. Rectal swab cultures before TRUSP-Bx provide useful information for selecting appropriate antimicrobials for prophylaxis and treatment of infection [31]. The American Urological Association Update Series also suggested extended coverage of fluoroquinolones in patients with comorbid conditions [32].

Transperineal prostate biopsy has also been addressed in the literature [27, 33, 34], and this route appears to be superior to the transrectal one in terms of bacteremia and UTI [10]. Thus, in this era of increasing microbial resistance, transperineal prostate biopsy may be a feasible alternative in order to minimize the risk of infection. Nevertheless, large comparison studies are necessary to elucidate the safety and efficacy of this modality.

In group B of the present study, we observed a sensitivity of over 80% to cefepime, piperacillin/tazobactam, amikacin, and ertapenem, while only 68.8% were sensitive to amoxicillin/clavulanate. However, 25% of the *E. coli* strains were resistant to second and third generations of cephalosporin. The resistance patterns we reported appeared worse than those previously published with fluoroquinolone prophylaxis [9, 20, 21]. Second and third generation cephalosporin, amoxicillin/clavulanate, and cefazolin/gentamycin are not optimal choices for first-line treatment when culture results are still pending. On the other hand, amikacin and ertapenem are recommended for treating patients with suspected UTI after TRUSP-Bx with levofloxacin prophylaxis. Fluoroquinolone should not be used to treat infectious complications following TRUSP-Bx empirically.

## 5. Conclusions

To our knowledge, this is the largest retrospective study to evaluate the efficacy of levofloxacin and pipemidic acid based prophylaxis in TRUSP-Bx. We quantified the incidence of febrile UTI and also demonstrated resistance patterns under prophylaxis with fluoroquinolones or nonfluorinated quinolones. In addition, we provided annual resistance patterns of *E. coli* in our institute. Fluoroquinolones appear superior to nonfluorinated quinolones for the prophylaxis of men undergoing TRUSP-Bx. Thus, although the rate of antimicrobial resistance is on the rise in the literatures, fluoroquinolone might still be the drug of choice preceding TRUS-guided prostate biopsy. Periodic reassessment of the efficacy of fluoroquinolone prophylaxis is necessary. Amikacin and ertapenem are recommended for the treatment of patients with suspected febrile UTI following TRUSP-Bx.

## 6. Limitations

This is a retrospective study which compares different prophylactic antibiotics in different time periods. The information of recent antibiotic use before operation could not be obtained for evaluation. The addition of cefazolin is a potential confounder. The febrile UTI rate could also be underestimated if the patients were treated at another hospital.

## Figures and Tables

**Table 1:** Patient characteristics and clinical presentation of infectious complications.

Group	A (pipemidic acid based prophylaxis)	B (levofloxacin prophylaxis)	*P* value
Patient characteristics			
Patients (*n*)	1313	2381	
Age, yrs (mean ± SD)	67.8 ± 10.4	67.7 ± 9.8	0.47
PSA^a^, ng/mL (mean ± SD)	60.0 ± 432.2	70 ± 437.5	0.5
Sextant biopsy	732 (55.8%)	714 (30.0%)	<0.001
≥12 core biopsy	581 (44.2%)	1667 (70.0%)	<0.001
Outpatient clinic	562 (42.8%)	631 (26.5%)	<0.001
Prostate cancer	342 (26.0%)	759 (31.9%)	<0.001
Chronic inflammation	118 (9.0%)	82 (3.4%)	<0.001
Infectious complications	48 (3.7%)	25 (1.0%)	<0.001
Clinical presentations			
Post-op day (mean ± SD)	1.7 ± 1.7	1.2 ± 0.7	0.42
Pyuria (*n* ^b^, %)	22/38 (57.9%)	16/17 (94.1%)	0.006
Leukocytosis (*n* ^c^, %)	27/42 (64.3%)	10/19 (52.6%)	0.41
Prolonged hospitalization, days (mean ± SD)	6.4 ± 6.3	5.0 ± 3.4	0.31
Positive urine culture (*n* ^d^, %)	16/48 (33.3%)	15/25 (60%)	0.046
Positive blood culture (*n* ^e^, %)	24/48 (50%)	12/25 (48%)	1

^a^The median PSA level is 10.1 and 10.0 ng/mL in group A and group B, respectively.

^b^ *n*: the number of patients with pyuria/the number of patients receiving urine analyses.

^c^ *n*: the number of patients with leukocytosis/the number of patients receiving blood tests.

^d^ *n*: the number of patients with positive urine culture/the number of patients receiving urine culture.

^e^ *n*: the number of patients with positive blood culture/the number of patients receiving blood culture.

**Table 2:** The relationship between potential risk factors and febrile UTI rate assessed with multivariate logistic regression analysis.

Risk factor	Febrile UTI (%)		OR	95% CI	*P *
	+	−			
Greater PSA level^a^	2.1	2.0	0.97	0.56–1.49	0.72
Greater age^b^	2.0	2.1	0.91	0.59–1.58	0.90
≥12 core biopsy	2.1	1.9	0.61	0.25–1.48	0.27
Out-patient clinic	1.9	2.1	0.93	0.38–2.32	0.88
Prostate cancer	1.5	2.3	1.46	0.80–2.65	0.22
Chronic inflammation	2.5	2.0	1.07	0.42–2.75	0.89
Levofloxacin prophylaxis	1.0	3.7	3.74	2.26–6.20	<0.001

^a^Greater than median PSA level.

^b^Greater than median age.

**Table 3:** An overview of bacterial isolates from blood and urine cultures.

Group	A (pipimedic acid based prophylaxis)	B (levofloxacin prophylaxis)
	*n* = 29	*n* = 19
*Escherichia coli *	17 (58.6%)	16 (84.2%)
*Klebsiella pneumoniae *	5 (17.2%)	1 (5.3%)
*Pseudomonas aeruginosa *	2 (6.9%)	—
*Enterobacter cloacae *	1 (3.4%)	1 (5.3%)
*Stenotrophomonas maltophilia *	1 (3.4%)	—
Coagulase-negative *Staphylococcus *	1 (3.4%)	—
*Corynebacterium* species	1 (3.4%)	—
*Morganella morganii *	1 (3.4%)	—
*Serratia marcescens *	—	1 (5.3%)

**Table 4:** Susceptibility of *E. coli* to antibiotics in infectious complications and from 2002 to 2009.

	TRUS-P Bx		Whole institute	
	Group A	Group B	2002 to 2004	2005 to 2009
	*n* = 17	*n* = 16	*n* = 19711	*n* = 47252
Antibiotics	% Susceptible			

Ampicillin	17.6	6.3	20	21
Amoxicillin and Clavulanate	41.2	68.8	62	56
Piperacillin/ Tazobactam	100	87.5	91	87
Cefazolin	58.8	68.8	70	62
Cefuroxime	82.4	—	78	—
Cefmetazole	94.1	75	87	83
Cefotaxime	88.2	75	86	77
Ceftazidime	—	75	—	77
Cefepime	94.1	81.3	97	90
Imipenem	100	100	99	99
Gentamycin	100	62.5	73	74
Amikacin	100	100	98	98
Levofloxacin	76.5	12.5	75	72
